# Poor inter-observer agreement in anatomical classifications of infrapopliteal arterial disease due to mandatory selection of only one target artery

**DOI:** 10.1177/02841851221102788

**Published:** 2022-05-26

**Authors:** Karin Ludwigs, Manne Andersson, Åse A Johnsson, Joakim Nordanstig, Angelica Svalkvist, Mårten Falkenberg, Erik Baubeta

**Affiliations:** 1 Department of Radiology, Institute of Clinical Sciences, Sahlgrenska Academy, 156329 University of Gothenburg, Gothenburg, Sweden; 2 Section of Vascular Surgery, Surgical Clinic, 293183 Hallands Hospital, Halmstad, Sweden; 3 Department of Surgery, Division of Vascular Surgery, 59583 Ryhov County Hospital, Jonkoping, Sweden; 4 Department of Clinical and Experimental Medicine, Faculty of Health Sciences, 4566 Linköping University, Linkoping, Sweden; 5 Department of Radiology, Region Västra Götaland, 70712 Sahlgrenska University Hospital, Gothenburg, Sweden; 6 Department of Molecular and Clinical Medicine, Institute of Medicine, Sahlgrenska Academy, 156329 University of Gothenburg, Gothenburg, Sweden; 7 Department of Vascular Surgery, 56749 Sahlgrenska University Hospital, Gothenburg, Sweden; 8 Department of Medical Imaging and Biomedical Engineering, 56749 Sahlgrenska University Hospital, Gothenburg, Sweden; 9 Department of Radiation Physics, Institute of Clinical Sciences, Sahlgrenska Academy, 156329 University of Gothenburg, Gothenburg, Sweden; 10 Diagnostic Radiology, Department of Translational Medicine, 5193 Lund University, Skåne University Hospital, Malmö, Sweden

**Keywords:** Peripheral arterial disease, endovascular procedures, magnetic resonance angiography, digital subtraction angiography, anatomical classification, infrapopliteal

## Abstract

**Background:**

Established anatomical classifications of infrapopliteal arterial lesion severity are based on assessment of only one target artery, not including all infrapopliteal arteries although multivessel revascularization is common.

**Purpose:**

To investigate the reproducibility of one of these classifications and a new aggregated score.

**Material and Methods:**

A total of 68 patients undergoing endovascular infrapopliteal revascularization at Sahlgrenska University Hospital during 2008–2016 were included. Preoperative magnetic resonance angiographies (MRA) and digital subtraction angiographies (DSA) were evaluated by three blinded observers in random order, using the infrapopliteal TransAtlantic Inter-Society Consensus (TASC) II classification. An aggregated score, the Infrapopliteal Total Atherosclerotic Burden (I-TAB) score, including all infrapopliteal arteries, was constructed and used for comparison.

**Results:**

Inter-observer agreement on lesion severity for each evaluated artery was good; Krippendorff’s α for MRA 0.64–0.79 and DSA 0.66–0.84. Inter-observer agreement on TASC II grade, based on the selected target artery as stipulated, was poor; Krippendorff's α 0.14 (95% confidence interval [CI]=−0.05 to 0.30) for MRA and 0.48 (95% CI=0.33–0.61) for DSA. Inter-observer agreement for the new I-TAB score was good; Krippendorff's α 0.76 (95% CI=0.70–0.81) for MRA and 0.79 (95% CI=0.74–0.84) for DSA.

**Conclusion:**

Reproducible assessment of infrapopliteal lesion severity can be achieved for separate arteries with both MRA and DSA using the TASC II definitions. However, poor inter-observer agreement in selecting the target artery results in low reproducibility of the overall infrapopliteal TASC II grade. An aggregated score, such as I-TAB, results in less variability and may provide a more robust evaluation tool of atherosclerotic disease severity.

## Introduction

Chronic limb-threatening ischemia (CLTI) is an important health problem. The increasing global prevalence of CLTI and increased utilization of invasive revascularization procedures substantially impact already scarce healthcare resources (1). Therefore, management algorithms to predict which patients with CLTI who are likely to benefit from invasive treatment are important.

Symptomatic chronic lower limb occlusive disease can be treated with open surgery or endovascular revascularization (2). The choice of revascularization technique is influenced by clinical severity and lesion characteristics as graded by anatomical classification systems that have been developed and endorsed by experts and societies (3,4). For lower limb arterial lesions there are two globally recognized anatomical classifications: the TransAtlantic Inter-Society Consensus for the Management Arterial Disease (TASC II) (3) classification and the more recent Global Anatomic Staging System (GLASS) (4). Both are based on the number, length, and complexity of infrainguinal lesions, although TASC II is also applicable in the aorto-iliac region.

In the aorto-iliac and femoropopliteal arteries there is only one major route to the distal part of the limb; thus, classifications in these segments are fairly straightforward. But in the infrapopliteal vascular segment, the situation is more complicated, particularly for patients with CLTI. Infrapopliteal circulation depends on three parallel arteries: the anterior tibial (ATA), the posterior tibial (PTA), and the fibular arteries (FA), with the tibiofibular trunk (TFT) serving as the common origin of the two latter. Both the TASC II and the GLASS classifications require that one single target artery is selected and both stipulate that only the chosen target artery determines the anatomic severity grade of the leg.

Anatomical classifications have dual purposes. One is to guide clinical decision-making. Open surgery is commonly recommended for longer lesions and more advanced arterial disease, while endovascular treatment is usually recommended for shorter and/or less complex lesions (5). However, optimal revascularization may include more than one infrapopliteal target artery. This is particularly important in endovascular procedures where there might be an option for revascularization in more than one artery simultaneously. Unfortunately, the anatomical classifications do not address this large subgroup of patients.

The second purpose of anatomical classifications is scientific: to enable accurate evaluation of different revascularization techniques by controlling for lesion severity in non-randomized trial designs. Despite the routine use of TASC II in many vascular registries, such as the Swedish National Registry for Vascular Surgery, Swedvasc, it is unknown if this anatomical evaluation is reproducible among clinicians. In a previous study, we investigated the agreement on infrapopliteal lesions between two imaging modalities, magnetic resonance angiography (MRA) versus digital subtraction angiography (DSA). We found good agreement between modalities but observed a large inter-observer variability regarding the choice of a target artery (6). If the choice of target artery has low reproducibility, then the scientific value of comparative studies based on the established classifications will also be low.

The aim of the present study was to compare the reproducibility of the infrapopliteal TASC II classification system with a suggested new score, the Infrapopliteal Total Atherosclerotic Burden (I-TAB), constructed by the aggregated lesion severity in all four infrapopliteal arteries. We hypothesized that a summation score is more reproducible than the established single-artery classifications and therefore might be a better tool from a clinical and a scientific perspective.

## Material and Methods

### Patients and images

All patients treated with isolated infrapopliteal endovascular revascularization for CLTI at Sahlgrenska University Hospital in Sweden during 2008–2016 were eligible for the study and identified in the Swedish National Registry for Vascular Surgery (Swedvasc). CLTI was defined as ischemic rest pain and/or non-healing ulcer or gangrene (Rutherford 4–6) (7). Patients aged <50 years were excluded (to avoid contamination of the study population with other underlying vascular diseases than atherosclerosis) as well as patients lacking infrapopliteal imaging with preoperative MRA and perioperative DSA. DSA is not routinely performed preoperatively; therefore, patients with open surgery were not included in this study.

Preoperative MRA and perioperative DSA images were obtained from the picture archiving and communication system. All MRA examinations were performed with 1.5-T magnetic resonance imaging (MRI) scanners. The majority of the examinations (52/68) were performed at the University hospital using a whole-body Philips MRI scanner. A minority (n = 16) were performed at neighboring hospitals. Standard gadolinium-based contrast protocols (gadoterate meglumine, Dotarem®; Guerbet, Roissy CDG, France, or, in some early examinations, gadobutrol, Gadovist®; Bayer Inc., Toronto, ON, Canada) were used with volumes of 10, 8, and 12 mL for the pelvis, thigh, and lower leg, respectively (6). All DSA images were retrieved from the original image acquisitions during the actual revascularization procedure using Siemens Artis Zeego angiography equipment. Images were obtained either with contrast from the introducer in the common femoral artery or with a catheter in the popliteal region, at the discretion of the operator.

### TASC II image evaluations

Four observers with 5–25 years of experience in evaluating vascular images assessed the images. One vascular surgeon and one radiologist evaluated all MRA and DSA scans. Additionally, two vascular surgeons participated, one observer evaluating all MRA scans and the other all DSA scans. In this way, three observers evaluated each modality. A consensus meeting was held before the evaluation began in which six patients not included in the actual study were assessed, to discuss the interpretation of the TASC II classification and establish a common evaluation strategy.

For the evaluation, an in-house developed image viewer software, ViewDEX, was used, and anonymized images were evaluated in a random order (8,9). The evaluations took place in a room with soft ambient light using Digital Imaging and Communications in Medicine (DICOM) calibrated high-quality medical display monitors. Three-dimensional maximum intensity projection (MIP) and dynamic MIP-series from MRA and conventional perioperative DSA images were presented to the observers. The observers were asked, by the ViewDEX software, to grade lesions according to the TASC II classification for each of the four individual infrapopliteal arteries and finally make an infrapopliteal TASC II grading based on the preferred target artery, as stipulated in the classification (3).

### I-TAB score image evaluations

A novel score was developed to determine inter-observer coherence in grading based on one single target artery (as in TASC II) compared with grading based on lesion severity in all the infrapopliteal arteries. To allow mathematical summation of observed lesions, the TASC II grades were converted to an integer where TASC A equals 1, B = 2, C = 3, and D = 4. Arteries without any lesion were scored 0. The range of values was 0–4 for ATA, PTA, and FA while the range of the shorter TFT was set to 0–3 (TASC D requires a total lesion length >10 cm). Thus, the suggested I-TAB is a mathematical summation of the TASC II scores for all four infrapopliteal arteries with the theoretical range of I-TAB from 0 (no infrapopliteal lesions) to 15 (long occlusions in all infrapopliteal arteries).

### Statistics

Krippendorff's α was used for statistical analysis by calculating image evaluation agreement. Krippendorff's α operates calculations of ordinal data with multiple observers and has the ability to manage missing data (10). The range of Krippendorff's α is between −1 to 1 with 0 as pure chance and 1 as perfect agreement. Values of Krippendorff's α >0.8 is considered to show reliable agreement and values of Krippendorff's α in the range of 0.667–0.8 imply tentative conclusions of agreement. The statistical software used was SPSS Statistics version 26.0 (IBM Corp., Armonk, NY, USA).

### Ethics

Individual consent was not required for this retrospective study. The ethics committee of the University granted ethical approval (reference nos. 220-17 [2017-04-26] and T1060-17 [2017-12-04].

## Results

### Patient population

**Fig. 1.**
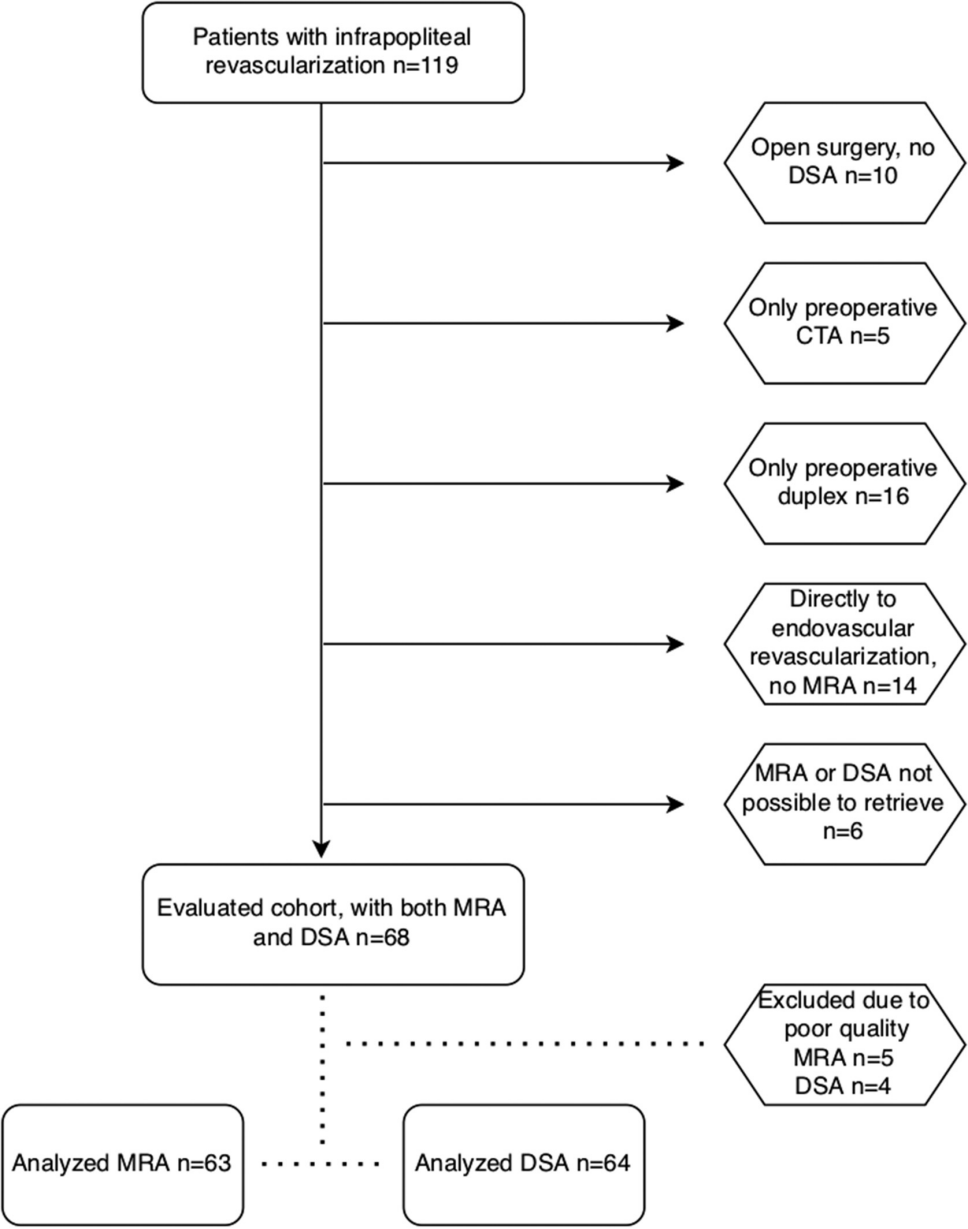
Flow chart showing the study population selection process. CTA, computed tomography angiography; DSA, digital subtraction angiography; MRA, magnetic resonance angiography.

During the study period, 119 patients with CLTI treated with infrapopliteal revascularization at Sahlgrenska University Hospital were identified in Swedvasc. Of these, 68 patients had both MRA and DSA images and were included in the study. After assessing images, we excluded images with too-low image quality (two or more observers were not able to grade the images according to TASC II due to poor image quality). Finally, 63 patients were included in the analysis of MRA, and 64 patients were included in the analysis of DSA. The study population selection process is displayed in Fig. 1.

### Arterial lesion agreement

**Fig. 2.**
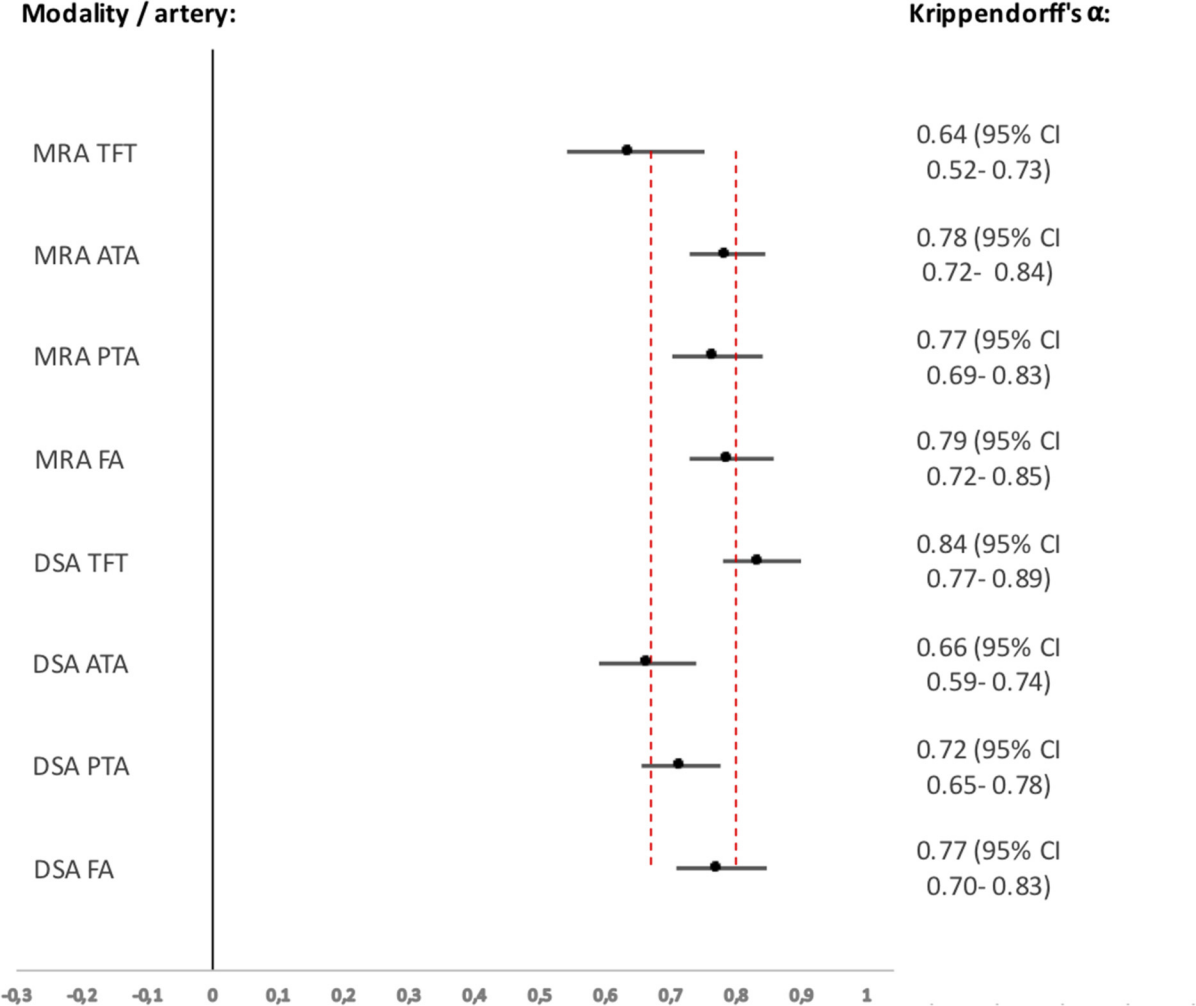
Agreement of TASC II for each infrapopliteal artery with Krippendorff's α. Krippendorff's α values of each artery for both imaging modalities, in relation to the threshold lines for tentative and reliable conclusions are set at 0.67 and 0.80, respectively. Values and 95% confidence intervals are shown to the right. ATA, anterior tibial artery; DSA, digital subtraction angiography; FA, fibular artery; MRA, magnetic resonance angiography; PTA, posterior tibial artery; TASC, TransAtlantic Inter-Society Consensus; TFT, tibiofibular trunk.

The inter-observer agreement on lesion severity in each separate artery based on MRA and DSA images evaluations was good, Krippendorff’s α 0.64–0.79 and 0.66–0.84, respectively (Fig. 2).

**Fig. 3.**
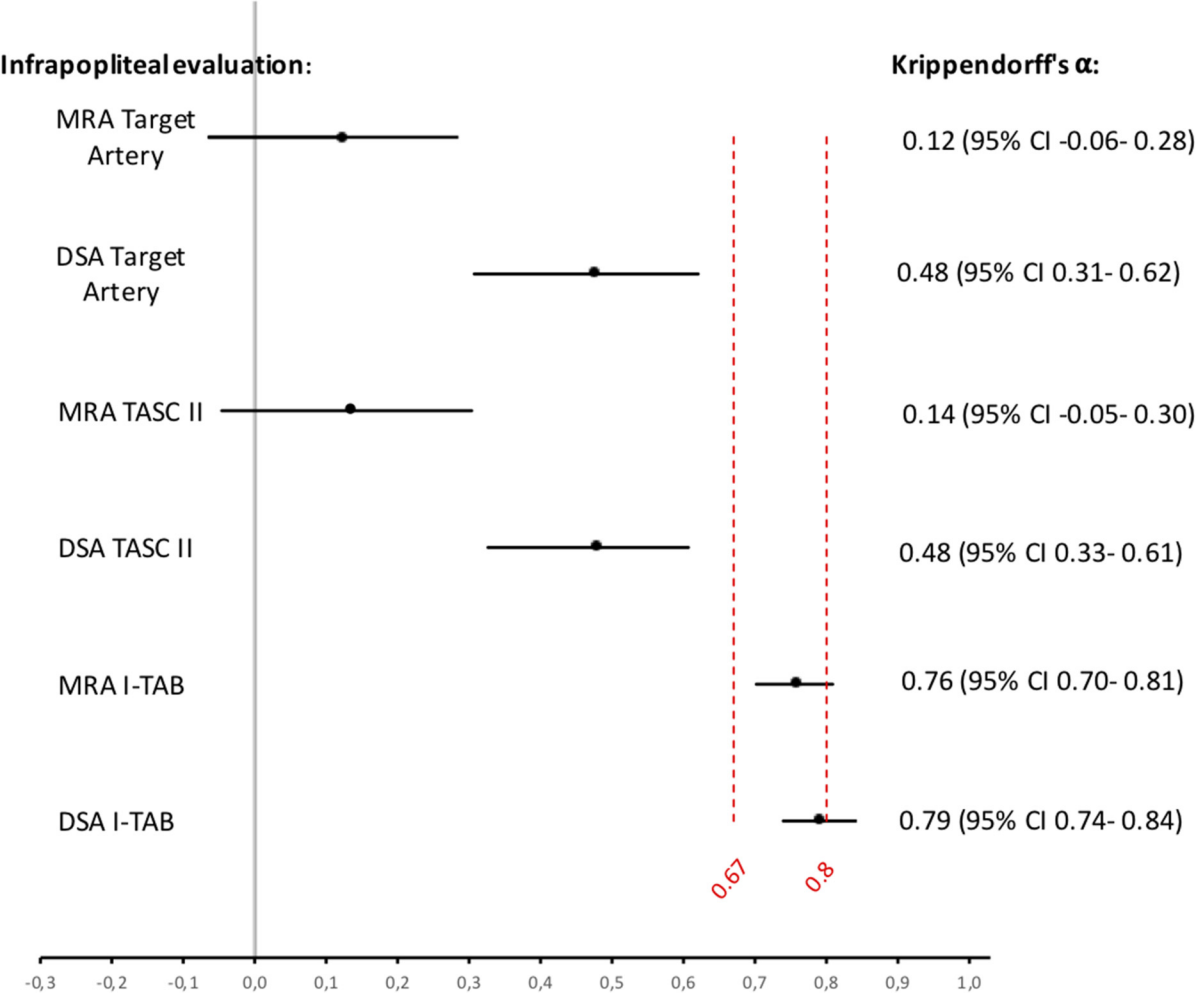
Agreement of infrapopliteal arterial disease with Krippendorff's α. Krippendorff's α values for choice of target artery, TASC II and I-TAB for both imaging modalities, in relation to the threshold lines for tentative and reliable conclusions are set at 0.67 and 0.80, respectively. Values and 95% confidence intervals are shown to the right. DSA, digital subtraction angiography; I-TAB, Infrapopliteal Total Atherosclerotic Burden; MRA, magnetic resonance angiography; TASC, TransAtlantic Inter-Society Consensus.

In terms of selecting an infrapopliteal target artery, the inter-observer agreement was poor, with a Krippendorff's α of 0.12 (95% confidence interval [CI] −0.06 to 0.28) for MRA and 0.48 (95% CI = 0.31–0.62) for DSA. The agreement for evaluation of formal TASC II, based on the chosen target vessel was also low, Krippendorff's α of 0.14 (95% CI = −0.05 to 0.30) for MRA and 0.48 (95% CI = 0.33–0.61) for DSA (Fig. 3).

The inter-observer agreement for classifying disease severity according to the I-TAB score (i.e. representing the total atherosclerotic burden in the infrapopliteal arteries) was higher for both imaging modalities. The Krippendorff's α value for MRA was 0.76 (95% CI = 0.70–0.81) and for DSA 0.79 (95% CI = 0.74–0.84) with narrower CIs (Fig. 3).

**Fig. 4.**
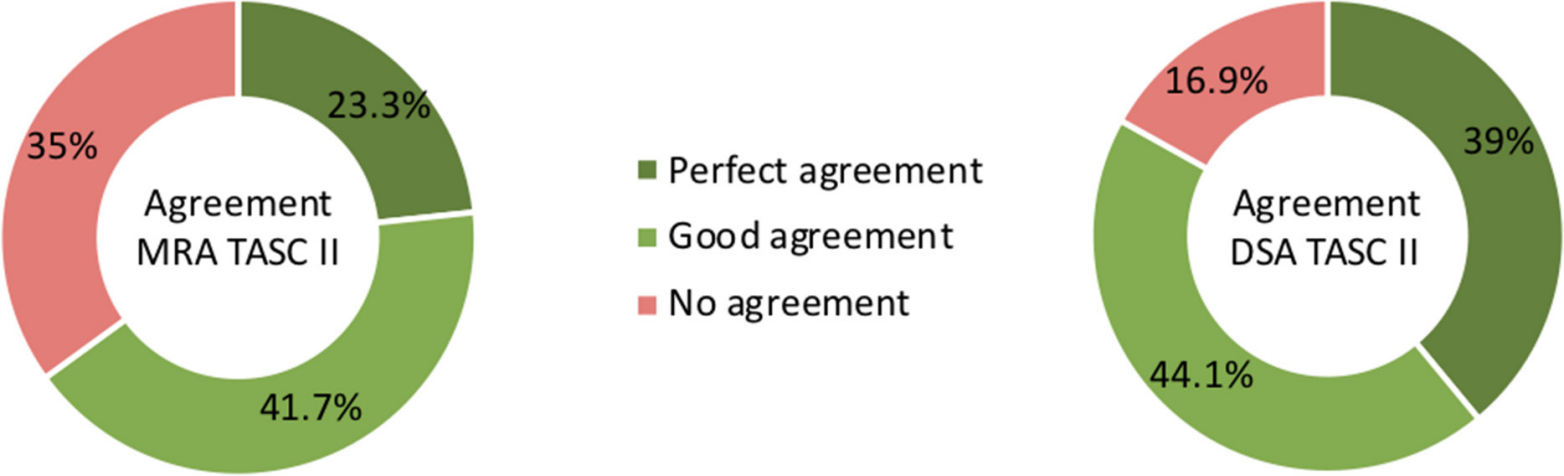
Percentage agreement between the three observers regarding MRA infrapopliteal TASC II and DSA infrapopliteal TASC II: perfect agreement = exactly the same evaluations for all three observers; good agreement = two observers with the same evaluation and one observer one TASC II grade away; and no agreement = all three observers scored differently. DSA, digital subtraction angiography; MRA, magnetic resonance angiography; TASC = TransAtlantic Inter-Society Consensus.

**Fig. 5.**
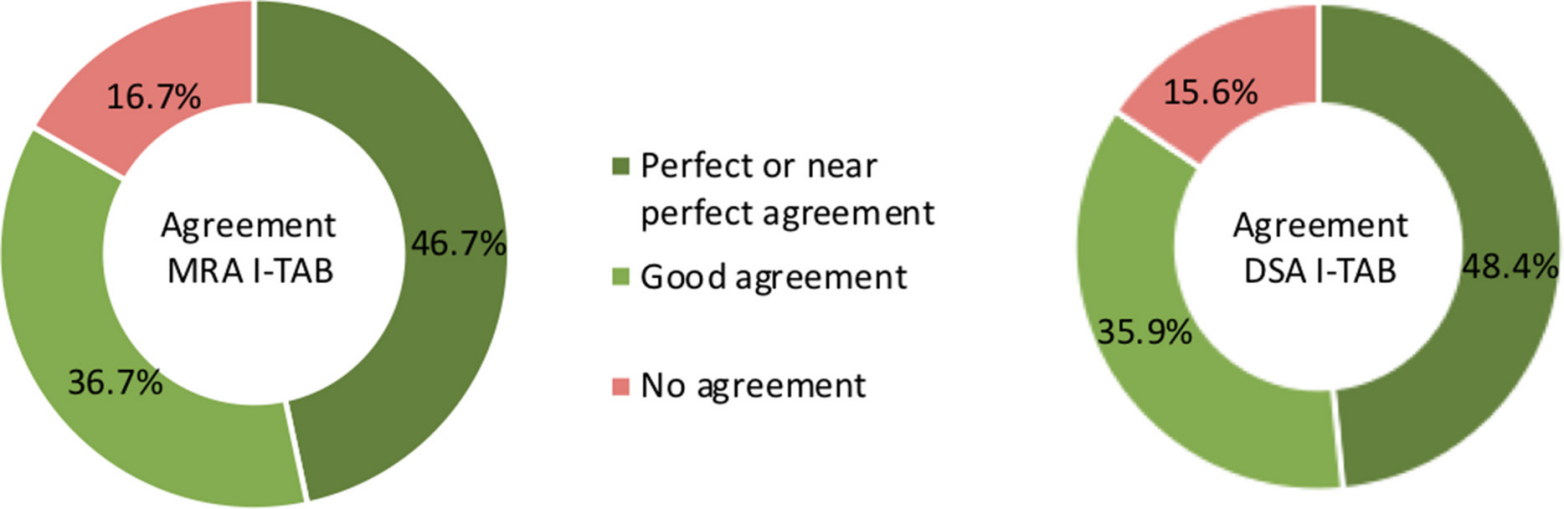
Percentage agreement between the three observers regarding MRA I-TAB and DSA I-TAB: perfect agreement = exactly the same evaluations for all three observers; near perfect agreement = one observer was one TASC II grade away; good agreement = two observers agreed, and the third observer was two TASC II grades away, or all three observers disagreed but only totally two TASC II grades away from each other; no agreement = observers with less agreement than above. DSA, digital subtraction angiography; I-TAB, Infrapopliteal Total Atherosclerotic Burden; MRA, magnetic resonance angiography.

The inter-observer agreement for both TASC II class and I-TAB score are illustrated in Figs. 4 and 5. For I-TAB the numerical range of possible scores is larger (range = 0–15), and it is therefore more challenging to achieve perfect agreement. Only 11.7% (MRA) and 15.6% (DSA) of the evaluations had a perfect agreement with all three observers.

## Discussion

This study of infrapopliteal arterial lesion assessment has three main findings. First, the agreement on TASC II criteria in each predetermined artery was good, with conclusive or reliable agreement between observers. Second, infrapopliteal anatomic lesion class based on one single target artery had poor inter-observer reproducibility due to low agreement on the choice of target artery. Third, an aggregated score, taking into account all infrapopliteal arteries, was more reproducible.

We used the TASC II classification for artery disease evaluation, as this was the main classification available when the study was initiated and as TASC II is noted in Swedvasc before revascularization. In 2019, the new GLASS classification was launched. However, both the TASC II and GLASS classifications require that a single target artery is selected for infrapopliteal assessment. This introduces several inherent limitations applicable to both classifications, from a clinical and a scientific perspective. First, different observers and operators may choose different target arteries, making comparison difficult to interpret over time and between clinicians. Second, particularly in endovascular procedures, more than one target artery may be revascularized, making the choice of one single target artery inadequate. In our cohort, almost one-third (29%) of the legs were eventually revascularized in more than one infrapopliteal artery (6). It is not clear if revascularization of one single infrapopliteal artery is equally effective in achieving limb salvage as revascularization of two or more arteries in cases where this is an option. A recent review article suggested that one artery is enough for wound healing (11). In contrast, a randomized study (not included in the review mentioned above) showed a benefit of multivessel revascularization with better wound healing but only a non-significant trend towards better limb salvage (12). Third, the choice of a target artery is influenced by the choice of revascularization technique, open or endovascular, potentially hampering comparisons of results between strategies. Indeed, the choice of target artery was so poor it approached the likelihood of pure chance.

**Fig. 6.**
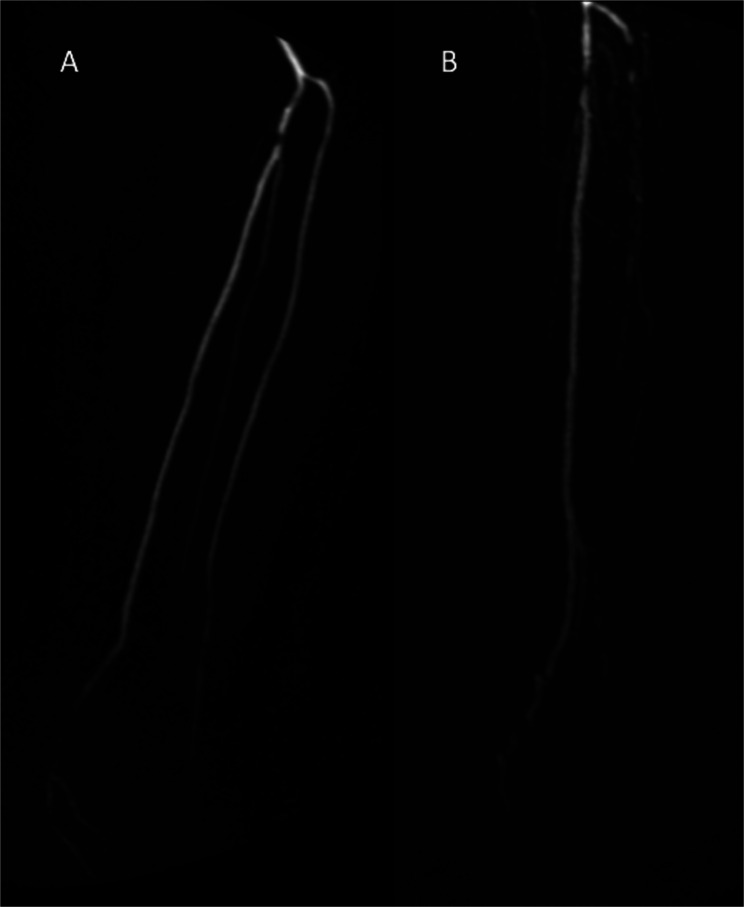
Illustration of TASC and I-TAB from our patient cohort. Patient A was evaluated as TASC A/I-TAB 4 and patient B was evaluated as TASC A/I-TAB 9. Though TASC remains the same, since the target artery is classified as TASC A, the atherosclerotic burden is quite different between these two patients. I-TAB, Infrapopliteal Total Atherosclerotic Burden; TASC, TransAtlantic Inter-Society Consensus.

It would be of value if preoperative assessment of infrapopliteal arteries is done in a more reproducible way. We found that an aggregated score that summarizes the overall lesion burden in all infrapopliteal arteries, the suggested I-TAB, was more reproducible than a single infrapopliteal artery classification system. When using I-TAB, the agreement between observers clearly improved. This indicates that an aggregated preoperative score may be a better tool for estimating lesion severity in patients with CLTI due to infrapopliteal lesions. The difference between TASC and I-TAB is illustrated in Fig. 6. The two patients scored TASC A but I-TAB increased with more widespread atherosclerotic disease. To be clinically and scientifically relevant, an aggregated score such as I-TAB, must be evaluated for its ability to predict the revascularization outcome, and this remains to be done. On the other hand, this also holds true for the internationally established infrapopliteal TASC II and GLASS classifications.

The present study has some limitations. First, the retrospective single-center design renders the results potentially sensitive to selection bias and may be subject to confounding. Another limitation is that the GLASS system, recently endorsed by several societies, was not yet published at the time of our analysis and hence was not used in the study. However, both the TASC II and the GLASS classification systems share the critical prerequisite that we address, i.e. that only one chosen target artery determines the final score. The study's strength is the structured reviewing process with anonymized images assessed in random order with dedicated software, where observers were blinded to patients’ characteristics as well as their own and the other observers’ assessments, and the inclusion of consecutive patients with CLTI.

In conclusion, we found that infrapopliteal arterial lesions in CLTI patients can be classified with good reproducibility with both MRA and DSA. However, inter-observer variability in the choice of target artery is a major concern leading to poor reproducibility when using the TASC II anatomical classification system. The proposed I-TAB scoring system, which summarizes the atherosclerotic burden in all infrapopliteal arteries, is more reproducible and may be a better tool for clinical decisions and scientific evaluations.

## References

[1] Sampson UKA Fowkes FGR McDermott MM, et al. Global and regional burden of death and disability from peripheral artery disease: 21 world regions, 1990 to 2010. Glob Heart 2014;9:145–158. 25432124 10.1016/j.gheart.2013.12.008

[2] Farber A Eberhardt RT. The current state of critical limb ischemia: a systematic review. JAMA Surg 2016;151:1070–1077. 27551978 10.1001/jamasurg.2016.2018

[3] Jaff MR White CJ Hiatt WR, et al. An update on methods for revascularization and expansion of the TASC lesion classification to include below-the-knee arteries: a supplement to the inter-society consensus for the management of peripheral arterial disease (TASC II): the TASC steering committee. Catheter Cardiovasc Interv 2015;86:611–625. 26256456 10.1002/ccd.26122

[4] Conte MS Bradbury AW Kolh P, et al. Global vascular guidelines on the management of chronic limb-threatening ischemia. J Vasc Surg 2019;69:3–125. 10.1016/j.jvs.2019.02.016 PMC8365864 31159978

[5] Aboyans V Ricco J-B Bartelink M-LEL, et al. 2017 ESC Guidelines on the Diagnosis and Treatment of Peripheral Arterial Diseases, in collaboration with the European Society for Vascular Surgery (ESVS): document covering atherosclerotic disease of extracranial carotid and vertebral, mesenteric, renal, upper and lower extremity arteries. Eur Heart J 2018;39:763–816. 28886620 10.1093/eurheartj/ehx095

[6] Fridh EB, Ludwigs K, Svalkvist A, et al. Comparison of magnetic resonans angiography and digital subtraction angiography for the assessment of infrapopliteal arterial occlusive lesions, based on the TASC II classification criteria. Diagnostics 2020;10:892. 10.3390/diagnostics10110892 PMC7693383 33142848

[7] Rutherford RB Baker JB Ernst C, et al. Recommended standards for reports dealing with lower extremity ischemia: revised version. J Vasc Surg 1997;26:517–538. 9308598 10.1016/s0741-5214(97)70045-4

[8] Håkansson M, Svensson S, Zachrisson S, et al. VIEWDEX: an efficient and easy-to-use software for observer performance studies. Radiation Protection Dosimetry 2010;139:42–51. 10.1093/rpd/ncq057 20200105

[9] Svalkvist A, Svensson S, Håkansson M, et al. VIEWDEX: A Status Report. Radiat Prot Dosimetry 2016;169:38–45. 10.1093/rpd/ncv543 26822421

[10] Hayes AF Krippendorff K. Answering the call for a standard reliability measure for coding data. Commun Methods Meas 2007;1:77–89.

[11] Anand GM Conway AM Giangola G. Singel versus multiple vessel endovascular tibial artery revascularization for critical limb ischemia: a review of the literature. Int J Angiol 2020;29:175–179. 33132674 10.1055/s-0040-1714662 PMC7581462

[12] Biagioni RB Biagioni LC Nasser F, et al. Infrapopliteal angioplasty of one or more than one artery for critical limb ischemia: a randomized clinical trial. Eur J Vasc Endovasc Surg 2018;55:518–527. 29402670 10.1016/j.ejvs.2017.12.022

